# Emodin Attenuates LPS-Induced Acute Lung Injury by Inhibiting NLRP3 Inflammasome-Dependent Pyroptosis Signaling Pathway *In vitro* and *In vivo*

**DOI:** 10.1007/s10753-021-01581-1

**Published:** 2021-11-17

**Authors:** Yuhan Liu, Luorui Shang, Jiabin Zhou, Guangtao Pan, Fangyuan Zhou, Shenglan Yang

**Affiliations:** 1 Department of Integrated Traditional Chinese and Western Medicine, Union Hospital, Tongji Medical College, Huazhong University of Science and Technology, Wuhan, 430022 China; 2 Department of Neurosurgery, Union Hospital, Tongji Medical College, Huazhong University of Science and Technology, Wuhan, 430022 China

**Keywords:** emodin, acute lung injury, NLRP3 inflammasome, pyroptosis, LPS

## Abstract

Emodin, the effective component of the traditional Chinese medicine Dahuang, has anti-inflammatory effects. However, the protective effects and potential mechanisms of emodin are not clear. This study investigated the protective effects and potential mechanisms of emodin on lipopolysaccharide (LPS)-induced acute lung injury (ALI) *in vitro* and *in vivo*. *In vivo*, we designed an LPS-induced ALI rat model. *In vitro*, we chose the J774A.1 cell line to establish an inflammatory cellular model, and knocked down NOD-like receptor family pyrin domain containing 3 (NLRP3) using small interfering RNA. The mRNA and protein expression of NLRP3, a C-terminal caspase recruitment domain (ASC), caspase 1 (CASP1), and gasdermin D (GSDMD) in cells and lung tissues were detected by western blot and real-time quantitative polymerase chain reaction (PCR). The expression levels of interleukin 1 beta (IL-1β) and IL-18 in the serum and supernatant were determined by the enzyme-linked immunosorbent assay. The degree of pathological injury in lung tissue was evaluated by hematoxylin and eosin (H&E) staining. *In vitro*, we demonstrated that emodin could inhibit NLRP3 and then inhibit the expression of ASC, CASP1, GSDMD, IL-1β, and IL-18. *In vivo*, we confirmed that emodin had protective effects on LPS-induced ALI and inhibitory effects on NLRP3 inflammasome -dependent pyroptosis. Emodin showed excellent protective effects against LPS-induced ALI by regulating the NLRP3 inflammasome-dependent pyroptosis signaling pathway.

## INTRODUCTION

Acute lung injury (ALI) and acute respiratory distress syndrome (ARDS) are the main causes of hypoxic respiratory failure during the hospitalization period in adults with high morbidity and mortality [1]. The characteristics of ALI/ARDS are infiltration of inflammatory cells, imbalance of the inflammatory response, and excess production of inflammatory mediators in lung tissue, which finally induce destruction of the blood-alveolar barrier and pulmonary edema [2]. To date, there is no specific drug for the treatment of ALI, so it is important to explore novel drugs and targets that can effectively treat lung injury.

Pyroptosis is a newly discovered pro-inflammatory programmed cell death mode. When the body is infected, the NOD-like receptor family pyrin domain containing 3 (NLRP3) recruits pro-caspase-1 and apoptosis-associated speck-like protein containing a caspase-recruitment domain (ASC) to form the NLRP3 inflammasome. Then the NLRP3 inflammasome processes pro-caspase-1 (CASP1) into mature CASP1, which activates gasdermin D (GSDMD) to form holes in the cell membrane [3]. Finally, the cells undergo pyroptosis and a large number of inflammatory factors such as interleukin 18 (IL-18) and IL-1β are released, which trigger a severe inflammatory response [4]. The NLRP3 plays an important role in the occurrence and progression of various systemic diseases, such as ALI [5], atherosclerosis [6], and inflammatory bowel disease [7]. Inhibition of NLRP3 inflammasome can alleviate ALI [8], indicating that the NLRP3 inflammasome may be a therapeutic target in ALI.

Emodin, a natural compound extracted from the traditional Chinese medicine rhubarb, has anti-inflammatory and anti-tumor properties [9]. Emodin can ameliorate lipopolysaccharide (LPS)-induced ALI [10, 11]. However, the protective mechanism of emodin against LPS-induced ALI is not clear. In this study, we showed that emodin may have protective effects on LPS-induced ALI by regulating the NLRP3 inflammasome-dependent pyroptosis signaling pathway.

## MATERIALS AND METHODS

### Reagents

Emodin was purchased from Meilun Biotech Co., Ltd. (Catalog No. MB5674; Dalian, China), and LPS was purchased from Sigma (Catalog No. Escherichia coli 055:B5; St. Louis, MO, USA). ChamQ™ SYBR® qPCR Master Mix (Catalog No. Q311-02) and HiScript® II Q RT SuperMix for quantitative PCR (qPCR) (Catalog No. R222-01) were obtained from Vazyme Biotechnology (Nanjing, China). The Cell Counting Kit-8 (CCK-8) was purchased from Dojindo Molecular Technologies (Kumamoto, Japan). Dexamethasone (DEX) was purchased from Xinxiang Changle Company (Xinxiang, China). Fetal bovine serum (FBS) was obtained from GIBCO (Grand Island, NY, USA) and Dulbecco’s modified Eagle’s medium (DMEM)/high glucose was purchased from HyClone (SH30022.01; Logan, UT, USA). The BCA Protein Assay Kit was obtained from Boster Biological Technology (Wuhan, China). The IL-1β Rat ELISA Kit was purchased from Elabscience Biotechnology (Catalog No. E-EL-R0012c; Wuhan, China), and the IL-18 Rat ELISA Kit was purchased from USCN (Catalog No. SEA064Ra; Wuhan, China). The IL-1β Mouse ELISA Kit was purchased from Boster Biological Technology (Catalog No. EK0394), and the IL-18 Mouse ELISA Kit was purchased from Elabscience Biotechnology (Catalog No. E-EL-M0730c). The Myeloperoxidase (MPO) Assay Kit (Catalog No. A044-1–1) and Malondialdehyde (MDA) Assay Kit (Catalog No. A003-1–2) were purchased from the Jiancheng Bioengineering Institute of Nanjing (Nanjing, Jiangsu, China). The Reactive Oxygen Species (ROS) Assay Kit was purchased from Beyotime (Wuhan, China).

### Cell Culture and Cytotoxicity of Emodin

The J774A.1 cell line was purchased from the China Center for Type Culture Collection (Hubei, China). J774A.1 cells were cultured in DMEM supplemented with 10% FBS. Cells were maintained at 37 °C in a humidified atmosphere containing 5% CO_2_/95% air. The CCK-8 assay was used to evaluate the cytotoxicity of emodin in J774A.1 cells. Briefly, cells were cultured overnight at a density of 5 × 10^3^ cells per well in 96-well plates. Then, emodin was added at different concentrations (10, 20, 40, 80, 160 μg/mL). After 24h, 10 μL CCK-8 reagent was added to each well and incubated for 1.5h in the dark. Then, the absorbance value of each well was measured on a microplate reader (Tecan Infinite F50; Mannedorf, Switzerland) at 450 nm.

### Establishment of Cellular Model and Intervention

J774A.1 cells were divided into six groups: control, LPS, emodin (20, 40, 80 μg/mL dissolved in dimethyl sulfoxide (DMSO) and diluted in phosphate-buffered saline (PBS) to a final DMSO concentration less than 0.1%), and DEX (0.5 μg/mL diluted in PBS) [12] groups. LPS was used to establish the cellular inflammatory model. Cells were cultured overnight at a density of 5 × 10^5^ cells per well in 6-well plates. The next day, fresh medium containing LPS (1 μg/mL) was added to all groups but the control group, and incubated for 2h. Then, DEX (0.5 μg/mL) and emodin (20, 40, 80 μg/mL) were added to the medium for 24h. The LPS group was treated with LPS only. After 24h, the cells were collected for qPCR and western blot analyses, and the supernatant was harvested for the enzyme-linked immunosorbent assay (ELISA).

### Small Interfering RNA-Mediated NLRP3 Knockdown in J774A.1 Cells

Mouse NLRP3 small interfering RNA (siRNA) (GGATGGCTTTGATGAGCTA) was designed and synthesized by Ruibo Biology (Guangzhou, China). J774A.1 cells were cultured in 6-well plates, and 50 nM siRNA duplexes were transfected using the riboFECT™ CP Transfection Kit (Guangzhou, China) according to the manufacturer’s instructions. The medium was changed after 8h. Then, the cells continued to be cultured for up to 48 or 72h. At 24h before harvest, the cells were pre-treated with LPS for 2h followed by treatment with emodin or DEX. Cells were collected at 48 and 72h after transfection for RNA and protein extraction.

### Animal Experiments

Male Sprague–Dawley rats (180–220 g) were purchased from the Animal Experimental Center, Tongji Medical College, Huazhong University of Science and Technology (Wuhan, China). These rats were maintained in a controlled environment at 23 °C with a 12h light/dark cycle for 1 week. Rats were randomly divided into six groups (*n* = 8 rats/group): normal, model (LPS only), LPS + emodin (20, 40, 80 mg/kg) [13], and LPS + DEX (1.8 mg/kg) groups. Rats in the emodin and DEX groups were intragastrically administered emodin (20, 40, and 80 mg/kg at concentrations of 1, 2, and 4 mg/mL) or DEX (1.8 mg/kg at a concentration of 90 μg/mL) for 30 min before establishment of the ALI model. Rats in the normal and model groups were intragastrically administered normal saline (0.9% NaCl). Then, all rats (except the normal group) were injected intraperitoneally with LPS (20 mg/kg). After 6h, the rats were sacrificed after being anesthetized with pentobarbital sodium (80 mg/kg), and the serum and lung tissues were collected for corresponding experiments.

### Specimen Collection

Blood tests and lung tissues were taken from the rats in all groups. Blood was collected in an anticoagulant test tube. After centrifugation at 3000 rpm (4 °C, 15 min), the serum was isolated and stored at −80 °C until use. The lung tissues were divided into three parts: the first part was used to determine the biochemical parameters, the second part was fixed in 4% paraformaldehyde and embedded in paraffin for hematoxylin and eosin (H&E) and immunohistochemistry (IHC) staining, and the remaining part was stored at –80 °C for extraction of RNA and protein.

### Oxidative Stress in the Lung

MPO and MDA activity in the lung tissues can reflect the degree of cell damage. According to the manufacturer’s instructions, 1 mL PBS was added to every 100 mg lung tissue, followed by grinding to a homogenate and centrifugation at 12,000 rpm for 10 min to collect the supernatant. Different working reagents were added to each well and incubated for the appropriate time. The optical density (OD) was measured at 460 nm for MPO and 532 nm for MDA.

### Western Blot Analysis

The lung tissues and J774A.1 cells were collected and lysed in RIPA buffer containing protease inhibitor cocktail by using an electric homogenate machine to obtain the supernatant. The protein concentration of each sample was detected by the BCA assay. Equal volumes of protein were resolved by 12% sodium dodecyl sulfate–polyacrylamide gel electrophoresis and electrotransferred to PVDF membranes (0.45 mm). The membranes were blocked in Tris-buffered saline containing 0.1% Tween 20 (TBST) and fat-free milk (5%) for 2h at room temperature. Then, the membranes were washed with TBST three times for 10 min and incubated with the following primary antibodies at 4 °C overnight: anti-NLRP3 (1:300, NBP2-12,446; Novus Biologicals, Littleton, CO, USA), anti-ASC (1:500, sc-514414; Santa Cruz, Dallas, TX, USA), anti-CASP1 (1:1000, 22,915–1-AP; Proteintech, Rosemont, IL, USA), anti-GSDMD (1:1000, A18281; ABclonel Science, Wuhan, China), and anti-GAPDH (1:1000, BM3864; Boster Biological). After washing three times with TBST for 10 min, the membranes were incubated with rabbit horseradish peroxidase (HRP)-conjugated secondary antibody at room temperature for 1h. The proteins were detected by enhanced chemiluminescence and analyzed by ImageJ software.

### Real-Time Quantitative PCR Analysis

Total RNA of the J774A.1 cells and lung tissues were isolated using Total RNA Extraction Reagent (No. R401-01; Vazyme, Nanjing, China) and then reverse-transcribed into cDNA with HiScript® II Q RT SuperMix according to the manufacturer’s instructions. The reaction conditions were established according to the manufacturer’s instructions (ChamQTM SYBR®qPCR Master Mix). Thermal cycling conditions were 30s at 95 °C, 5s at 95 °C, and 30s at 60 °C followed by 40 cycles, and at 95 °C for 15s, 60 °C for 1 min, and 95 °C for 15s in the StepOne Plus Real-Time PCR System (Applied Biosystems, Foster City, CA, USA). The primers were synthesized by Tsingke Biology (Wuhan, China). The sequences of all primers are listed in Table 1.

**Table 1:** Sequences of Primers for RT-PCR

Gene	Primer sequence	Rat (5′ to 3′)	Mouse (5′ to 3′)
NLRP3	Forward primer	TCTTTGCGGCTATGTACTATCT	ATTACCCGCCCGAGAAAGG
	Reverse primer	TTCTAATAGGACCTTCACGT	TCGCAGCAAAGATCCACACAG
ASC	Forward primer	ATCCTGGACGCTCTTGAAAACTT	CTTGTCAGGGGATGAACTCAAAA
	Reverse primer	GCTCCTGTATGCCCATGTCTCTA	GCCATACGACTCCAGATAGTAGC
CASP-1	Forward primer	CGGGCAAGCCAGATGTTTAT	ACAAGGCACGGGACCTATG
	Reverse primer	AACCACTCGGTCCAGGAAATG	TCCCAGTCAGTCCTGGAAATG
GSDMD	Forward primer	CCAACATCTCAGGGCCCCAT	CCATCGGCCTTTGAGAAAGTG
	Reverse primer	TGGCAAGTTTCTGCCCTGGA	ACACATGAATAACGGGGTTTCC
GAPDH	Forward primer	GACATGCCGCCTGGAGAAAC	AGGTCGGTGTGAACGGATTTG
	Reverse primer	AGCCCAGGATGCCCTTTAGT	TGTAGACCATGTAGTTGAGGTCA

### Measurement of ROS Production

J774A.1 cells were seeded into a 12-well plate (2.5 × 10^5^ cells/well) for 12h. Then, a cellular model was established and treated according to the above methods. Dichlorodi-hydrofluorescein diacetate (DCFH-DA) was diluted 1:1000 with serum-free medium. Then, the cell culture solution was removed, 2 mL diluted DVFH-DA was added to the cells and incubated at 37 °C for 30 min in the dark, and cells were washed twice with serum-free medium. Finally, the cells were observed by fluorescence microscopy (BX53; Olympus, Tokyo, Japan).

### Histopathologic Evaluation of the Lung Tissue

The lung tissues were soaked in 10% neutral buffered formalin for 24h. Then, samples were dehydrated in graded alcohol dilutions. Subsequently, we embedded the tissues in wax and sliced them. The paraffin sections were stained with H&E, and pathological changes in the lung tissues were observed with a light microscope (BX53; Olympus, Tokyo, Japan).

### IHC Analysis

The IHC procedure was as follows. The wax blocks of the embedded rat lung tissues were sliced and dewaxed. The sections were immersed in EDTA antigen repair buffer, heated in a microwave oven for 10 min at 100 °C, and washed three times with PBS for 3 min. Then, 3% hydrogen peroxide was added to the sections and incubated at room temperature for 15 min to block the endogenous peroxidase. Next, the sections were washed three times with PBS for 3 min and then blocked in normal goat serum blocking solution at room temperature for 20 min. After drying, the sections were incubated with primary antibodies (anti-NLRP3, 1:50; anti-ASC, 1:100; anti-CASP1, 1:200; anti-GSDMD, 1:100) at 4 °C overnight and washed three times with PBS for 3 min. Then, the sections were incubated with HRP-conjugated goat anti-rabbit IgG (1:500) at room temperature for 20 min and washed three times with PBS for 4 min. Freshly prepared DAB substrate solution was added dropwise to the sections and incubated for 3–15 min. The sections were washed, dyed, dehydrated, made transparent, sealed, and observed under an optical microscope (BX53; Olympus). The intensity of positive IHC staining was quantified by Image-Pro Plus software version 6.0.

### ELISA

The expression levels of IL-1β and IL-18 in the cell supernatant and rat serum were detected by ELISA kits. According to the manufacturer’s instructions, work reagents were added to each well in sequence, and the OD was detected at 450 nm in a microplate reader (Tecan Infinite F50; Mannedorf).

### Statistical Analyses

All statistical data were analyzed by GraphPad Prism software version 8.0. One-way analysis of variance with the post hoc Dunnett’s test was used to determine the significance of the statistical results. Data are expressed as the mean ± standard deviation (SD). *P* < 0.05 was considered statistically significant.

## RESULTS

### *In vitro* Cytotoxicity of Emodin

**Fig. 1:**
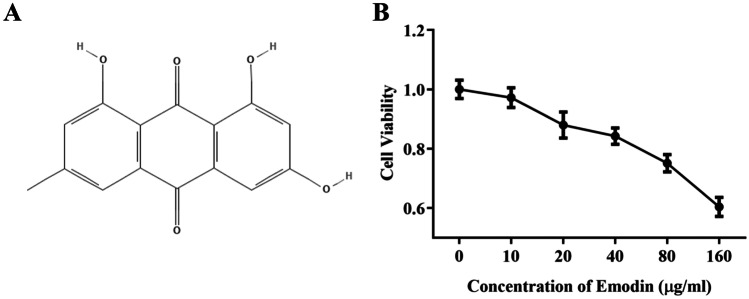
**A** The chemical structure of emodin. **B** A CCK-8 assay of J774A.1 cell viability after emodin treatment. Values are expressed as the mean ± SD of three individual experiments.

As shown in Fig. 1A, the molecular formula of emodin is C_15_H_10_O_5_. To assess the cytotoxicity of emodin on J774A.1 cells, we evaluated the viability of J774A.1 cells treated with different concentrations of emodin (10, 20, 40, 80, 160 μg/mL) after 24h according to the instructions of the CCK-8 kit. As shown in Fig. 1B, the concentration of emodin that led to 75% cell viability was 80 μg/mL; thus, we set the low concentration at 20 μg/mL, middle concentration at 40 μg/mL, and high concentration at 80 μg/mL.

### Effects of Emodin on ROS in LPS-Stimulated J774A.1 Cells

**Fig. 2:**
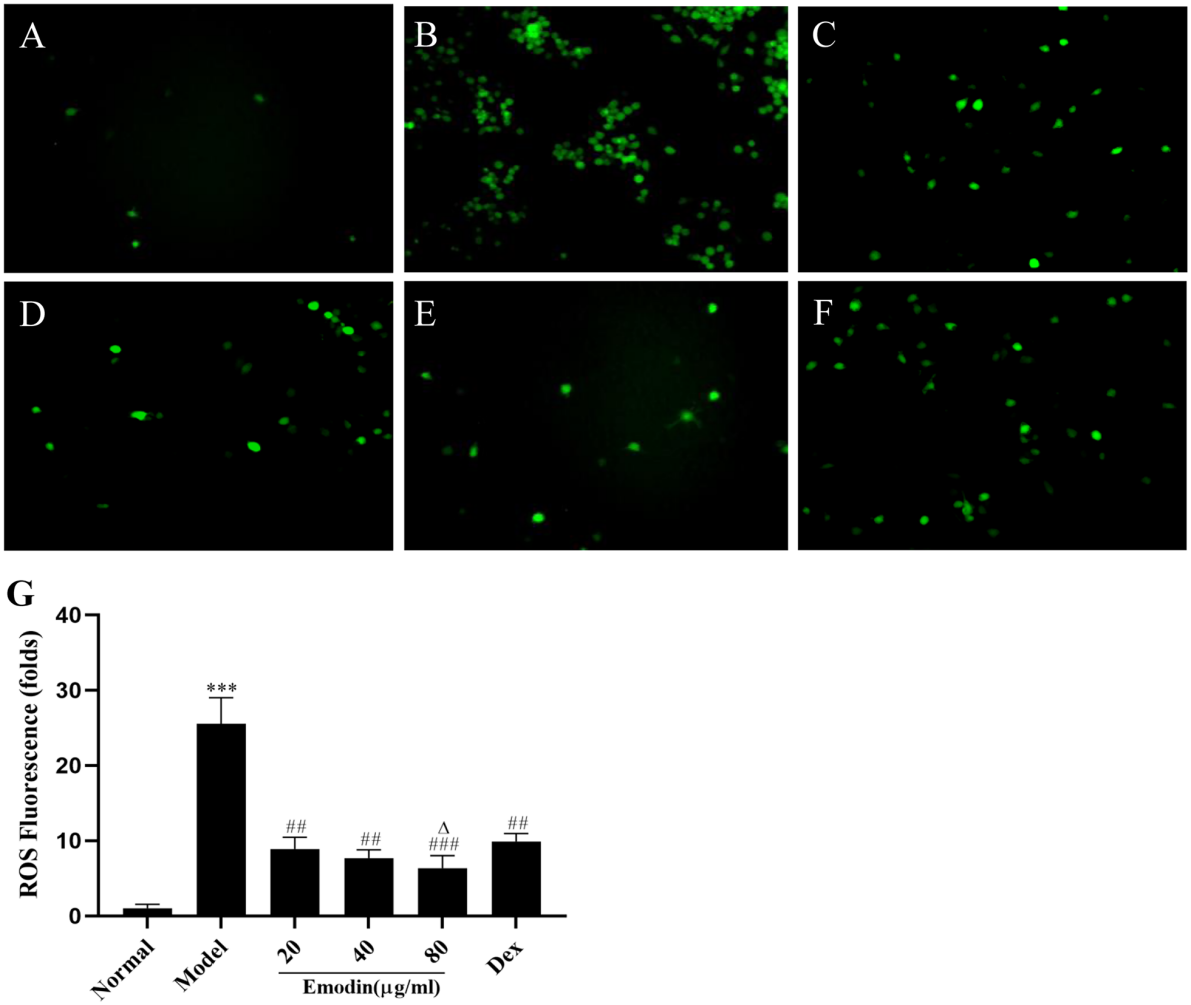
Effect of emodin on ROS in J774A.1 cells after LPS stimulation. **A** Normal group. **B** Model group. **C** Emodin (20 μg/mL) group. **D** Emodin (40 μg/mL) group. **E** Emodin (80 μg/mL) group. **F** DEX group. The images were captured using fluorescence microscopy at 400 × magnification. **G** ROS fluorescence intensities were analyzed using ImageJ and expressed in fold change. Values are expressed as the mean ± SD of three individual experiments. ^***^P < 0.001 vs. normal group. ^##^*P* < 0.01, ^###^*P* < 0.001 vs. model group; ^∆^*P* < 0.05 vs. DEX group.

ROS played an important role in the regulation of inflammation. An imbalance between the production and clearance of ROS can activate the NLRP3 inflammasome and lead to different degrees of tissue damage [14, 15]. We observed the production of ROS to evaluate the antioxidant effect of emodin. As shown in Fig. 2A-G, emodin suppressed the levels of ROS in LPS-stimulated J774A.1 cells.

### Effects of Emodin on NLRP3 Inflammasome-Dependent Pyroptosis Signaling Pathway in J774A.1 Cells After LPS Stimulation

**Fig. 3:**
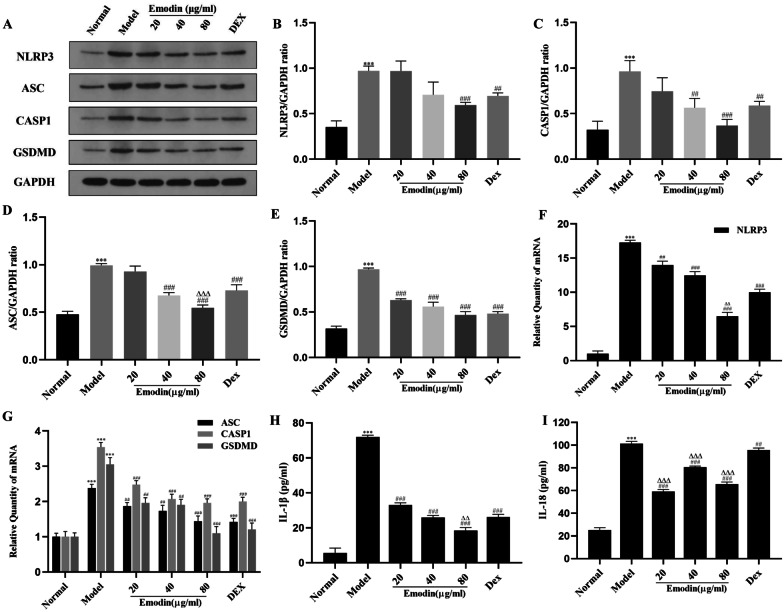
Effects of emodin on NLRP3 and downstream molecules in LPS-stimulated J774A.1 cells. **A**–**E** The protein levels of NLRP3, ASC, CASP1, and GSDMD were detected by western blotting, and the grayscale values of the bands were analyzed using ImageJ software. **F**, **G** The mRNA levels of NLRP3, CASP1, ASC, and GSDMD were detected by RT-PCR. **H**, **I** The expression of IL-1β and IL-18 in the cell supernatant was detected by ELISA. Values are expressed as the mean ± SD of three individual experiments. ^***^P < 0.001 vs. normal group. ^#^*P* < 0.05, ^##^*P* < 0.01, ^###^*P* < 0.001 vs. model group; ^∆∆^*P* < 0.01, ^∆∆∆^*P* < 0.001 vs. DEX group.

To assess the effects of emodin on the NLRP3 inflammasome *in vitro*, we used J774A.1 cells as the experimental model to detect the mRNA and protein expression of molecules related to NLRP3 inflammasome-dependent pyroptosis such as NLRP3, CASP1, ASC, and GSDMD. As shown in Fig. 3A-G, the mRNA and protein levels of NLRP3 in the model group were markedly increased compared with the normal group. Meanwhile, emodin and LPS had opposite effects on the expression of NLRP3. Moreover, the mRNA and protein expression levels of CASP1, ASC, and GSDMD were also markedly elevated in the model group. After intervention with different concentrations of emodin and DEX for 24h, the mRNA expression of NLRP3, CASP1, ASC, and GSDMD in the 20, 40, and 80 μg/mL emodin groups; the protein expression of NLRP3 in the 80 μg/mL emodin group; the protein expression of CASP1 and ASC in the 40 and 80 μg/mL emodin groups; and the protein expression of GSDMD in the 20, 40, and 80 μg/mL emodin groups were markedly decreased compared with the model group. More importantly, the mRNA levels of NLRP3 and the protein levels of ASC in the 80 μg/mL emodin group were lower than those in the DEX group. The effects of emodin on other molecules were similar to those of DEX. The above results showed that emodin significantly inhibited the NLRP3-dependent pyroptosis signaling pathway in J774A.1 cells after LPS stimulation.

### Effects of Emodin on IL-1β and IL-18 Production in LPS-Stimulated J774A.1 Cells

IL-1β and IL-18 are pivotal pro-inflammatory mediators in the NLRP3 inflammasome-dependent pyroptosis signaling pathway. To confirm the anti-inflammatory effects of emodin, we detected the levels of IL-1β and IL-18 in the cell supernatant by ELISA. As shown in Fig. 3H, I, the levels of IL-1β and IL-18 in the cell supernatant were significantly evaluated in the model group compared to the normal group, and the levels of IL-1β and IL-18 in the emodin groups were markedly decreased compared with the model groups. These results showed that emodin suppressed the production of IL-1β and IL-18 in LPS-stimulated J774A.1 cells.

### Effects of Emodin on the NLRP3 Inflammasome-Dependent Pyroptosis Signaling Pathway in J774A.1 Cells After NLRP3 Knockdown

**Fig. 4:**
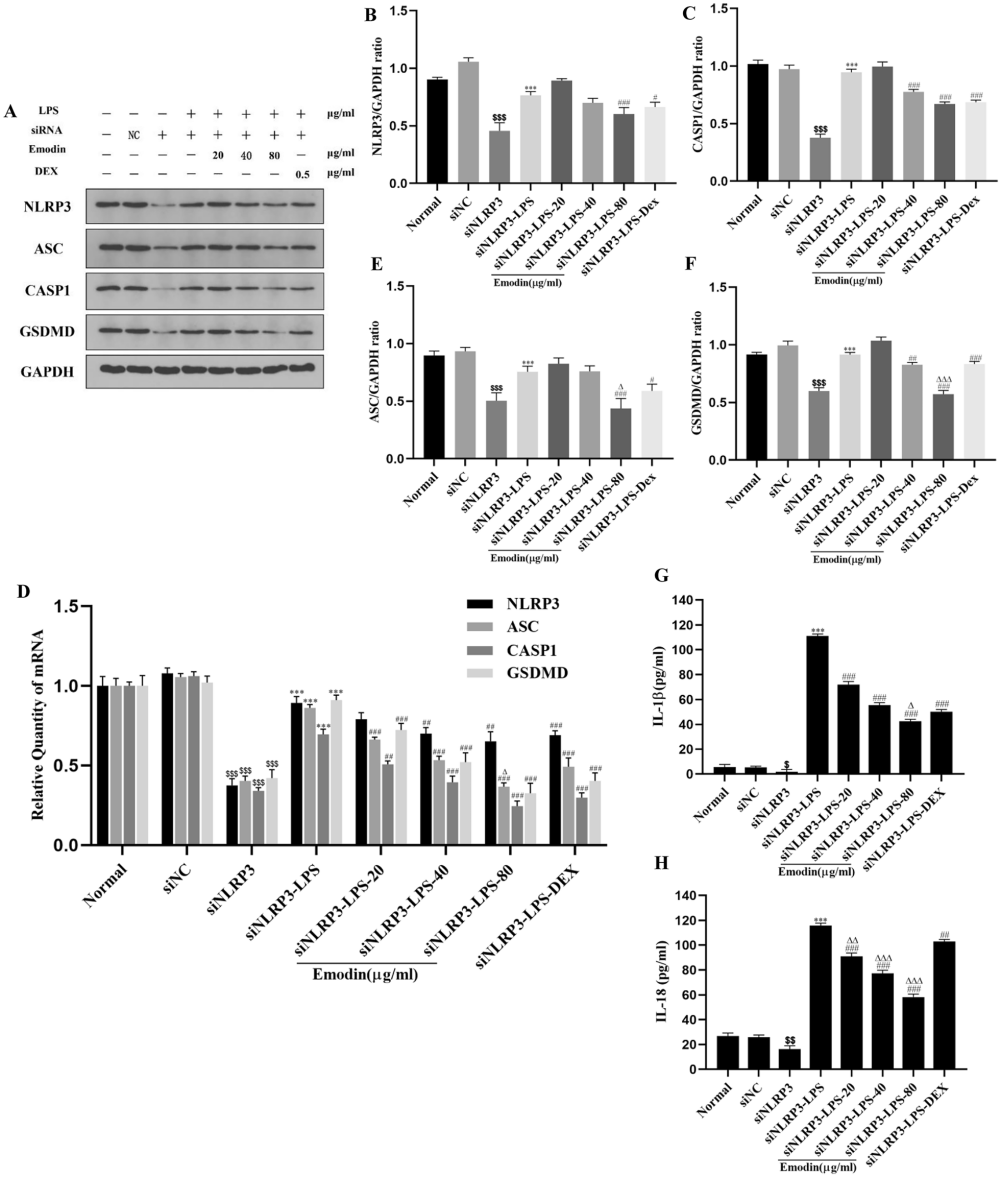
Effects of emodin on NLRP3 and downstream molecules in LPS-stimulated J774A.1 cells after NLRP3 knockdown. **A**, **D** The mRNA and protein levels of NLRP3, ASC, CASP1, and GSDMD in J774A.1 cells after transfection were detected by RT-PCR and western blotting. **B**, **C**, **E**, **F** The grayscale values of the bands were analyzed using ImageJ software. **G**, **H** The expression of IL-1β and IL-18 in the cell supernatant after transfection was detected by ELISA. Values are expressed as the mean ± SD of three individual experiments. ^$^*P* < 0.05, ^$$^*P* < 0.01, ^$$$^*P* < 0.01 vs. siNC group; ^***^P < 0.001 vs. siNLRP3 group; ^#^*P* < 0.05, ^##^*P* < 0.01, ^###^*P* < 0.001 vs. siNLRP3-LPS group; ^∆^*P* < 0.05, ^∆∆^*P* < 0.01, ^∆∆∆^*P* < 0.001 vs. siNLRP3-DEX group. siNLRP3: NLRP3 was knocked down in J774A.1 cells by siRNA.

We used siRNA to knock down NLRP3 in J774A.1 cells to downregulate its expression. The transfection procedure was the same as described above. As shown in Fig. 4A-F, there was no difference between the normal group and siRNA negative control (siNC) group. The mRNA and protein levels of NLRP3, CASP1, ASC, and GSDMD in the siNLRP3 group were significantly downregulated compared with the NC group. The levels of NLRP3 and its downstream molecules CASP1, ASC, and GSDMD in the siNLRP3-LPS group were increased compared with the siNLRP3 group. However, compared with the siNLRP3-LPS group, the mRNA levels of NLRP3 in the 40 and 80 μg/mL emodin groups and CASP1, ASC, and GSDMD in the 20, 40, and 80 μg/mL emodin groups were markedly suppressed. In addition, the protein levels of CASP1 and GSDMD in the 40 and 80 μg/mL emodin groups and NLRP3 and ASC in the 80 μg/mL emodin group were notably inhibited. In addition, the ASC mRNA level in the 80 μg/mL emodin group, as well as the protein levels of ASC and GSDMD in the 80 μg/mL emodin group, was markedly more reduced than those in the DEX group. These data showed that emodin inhibited the expression of NLRP3, and then inhibited the NLRP3-dependent pyroptosis signaling pathway.

### Effects of Emodin on IL-1β and IL-18 Production in LPS-Stimulated J774A.1 Cells After NLRP3 Knockdown

IL-1β and IL-18 are inflammatory products in the NLRP3 inflammasome-dependent pyroptosis signaling pathway, and their expression levels are regulated by the NLRP3 inflammasome. To determine whether emodin can reduce the production of IL-1β and IL-18 by inhibiting NLRP3, we used siRNA technology to silence NLRP3 in J774A.1 cells and detected the levels of IL-1β and IL-18 in the cell supernatant by ELISA. As shown in Fig. 4G, H, there was no difference between the normal group and siNC group. The levels of IL-1β and IL-18 in the siNLRP3 group were significantly downregulated compared with the siNC group. The levels of IL-1β and IL-18 in the siNLRP3-LPS group were increased compared with the siNLRP3 group. Compared with the siNLRP3-LPS group, the levels of IL-1β and IL-18 in the 20, 40, and 80 μg/mL emodin groups and DEX group were notably suppressed. Moreover, the levels of IL-1β in the 80 μg/mL emodin group and IL-18 in the 20, 40, and 80 μg/mL emodin groups were significantly decreased compared to the DEX group. These data showed that emodin inhibited NLRP3, and then suppressed the production of IL-1β and IL-18.

### Effects of Emodin on Lung Histopathology

**Fig. 5:**
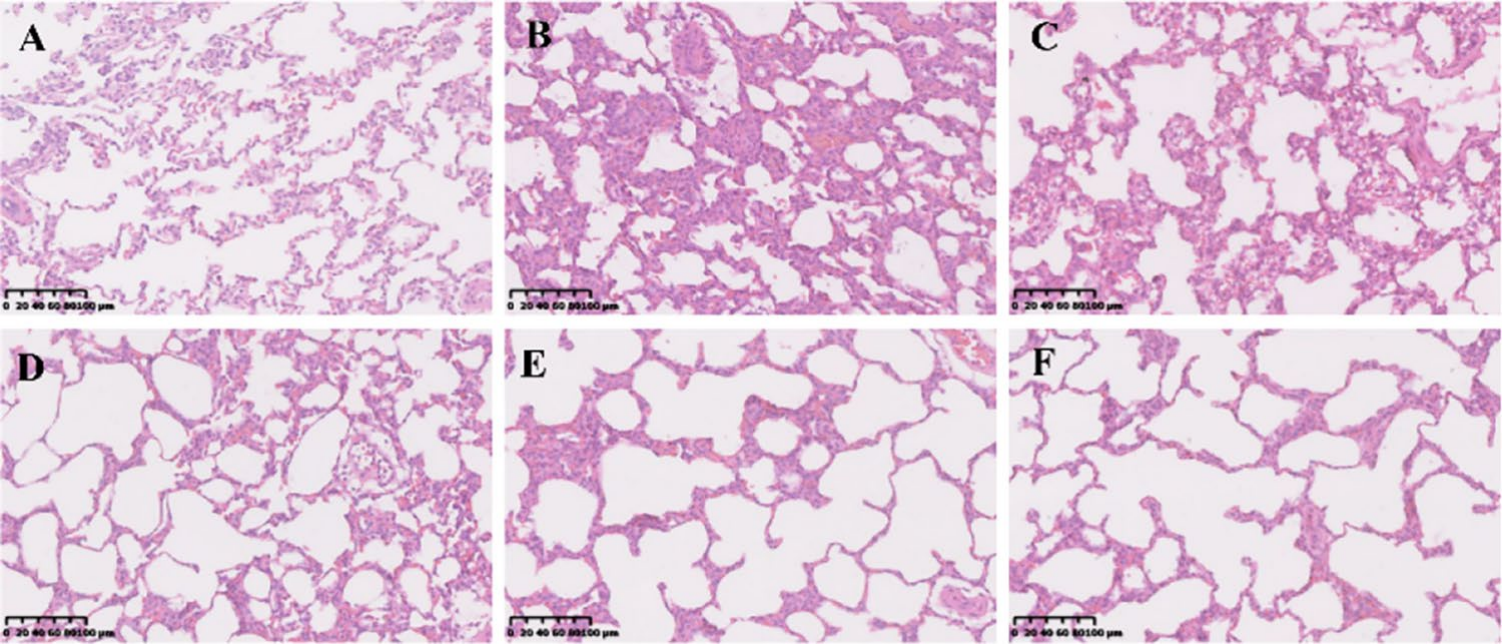
Pathological changes in the lung tissues were observed by H&E staining (original magnification 200 ×). **A** Normal group. **B** Model group. **C** Emodin (20 mg/kg) group. **D** Emodin (40 mg/kg) group. **E** Emodin (80 mg/kg) group. **F** DEX group. The lung tissues of rats treated with different concentrations of emodin and DEX looked healthier than those of the model rats.

To determine the effects of emodin on LPS-induced lung injury, the pathological changes in lung tissue were assessed by H&E staining. The pathological changes in lung tissues in the LPS group were characterized by the increased accumulation of inflammatory cells and alveolar hemorrhage (Fig. 5A-F). However, the LPS-induced severe pathological changes were weakened by treatment with emodin or DEX. The effects of a high concentration of emodin (80 mg/kg) on LPS-induced lung injury in rats were similar to those of DEX. These results suggested that emodin protected the lung from injury induced by LPS.

### Effects of Emodin on LPS-Induced MPO and MDA Levels in the Lung Tissues

**Fig. 6:**
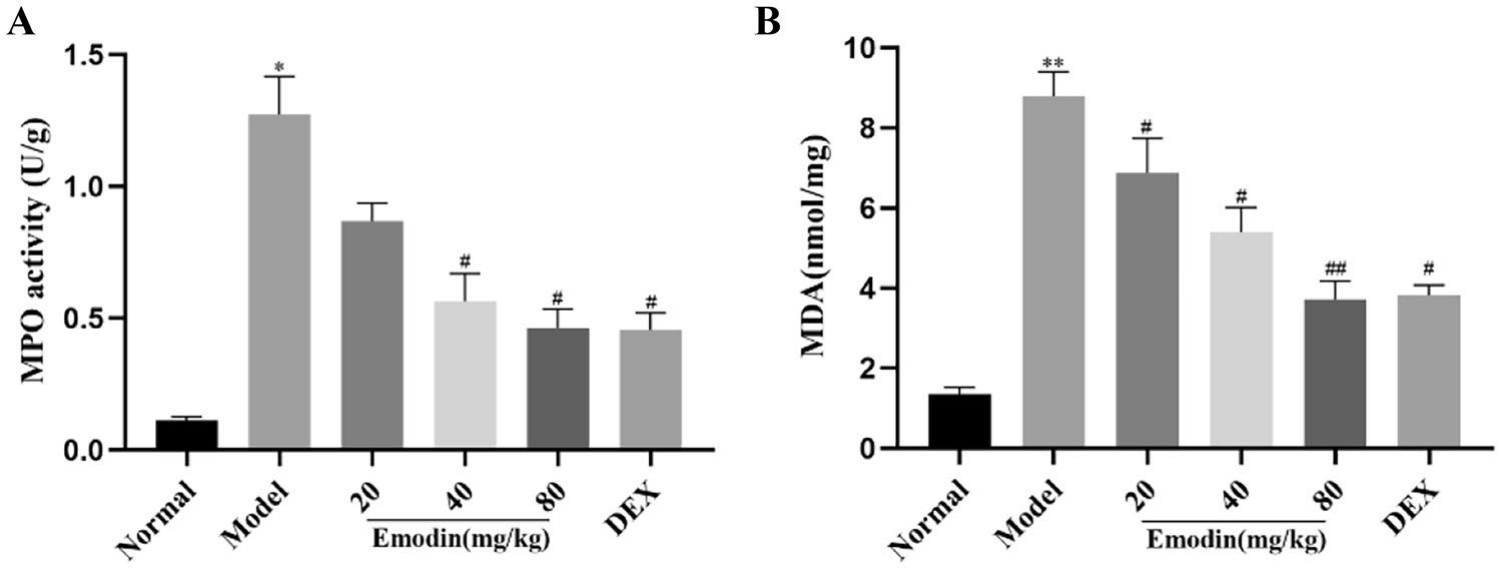
Effects of emodin on LPS-induced activity of MPO and MDA in the lung tissues. **A** MPO activity. **B** MDA activity in lung tissues. All data are presented as the mean ± SD (*n* = 8). ^*^P < 0.05, ^**^P < 0.01 vs. normal group. ^#^*P* < 0.05, ^##^*P* < 0.01 vs. model group.

The MPO and MDA levels in the lung tissues of the LPS group were notably higher than those in the normal group (Fig. 6A, B). Emodin and DEX both reduced MPO and MDA. These results showed that emodin suppressed lipid peroxidation and alleviated LPS-induced ALI in rats.

### Effects of Emodin on the NLRP3 Inflammasome-Dependent Pyroptosis Signaling Pathway in Rat Lung Tissues

**Fig. 7:**
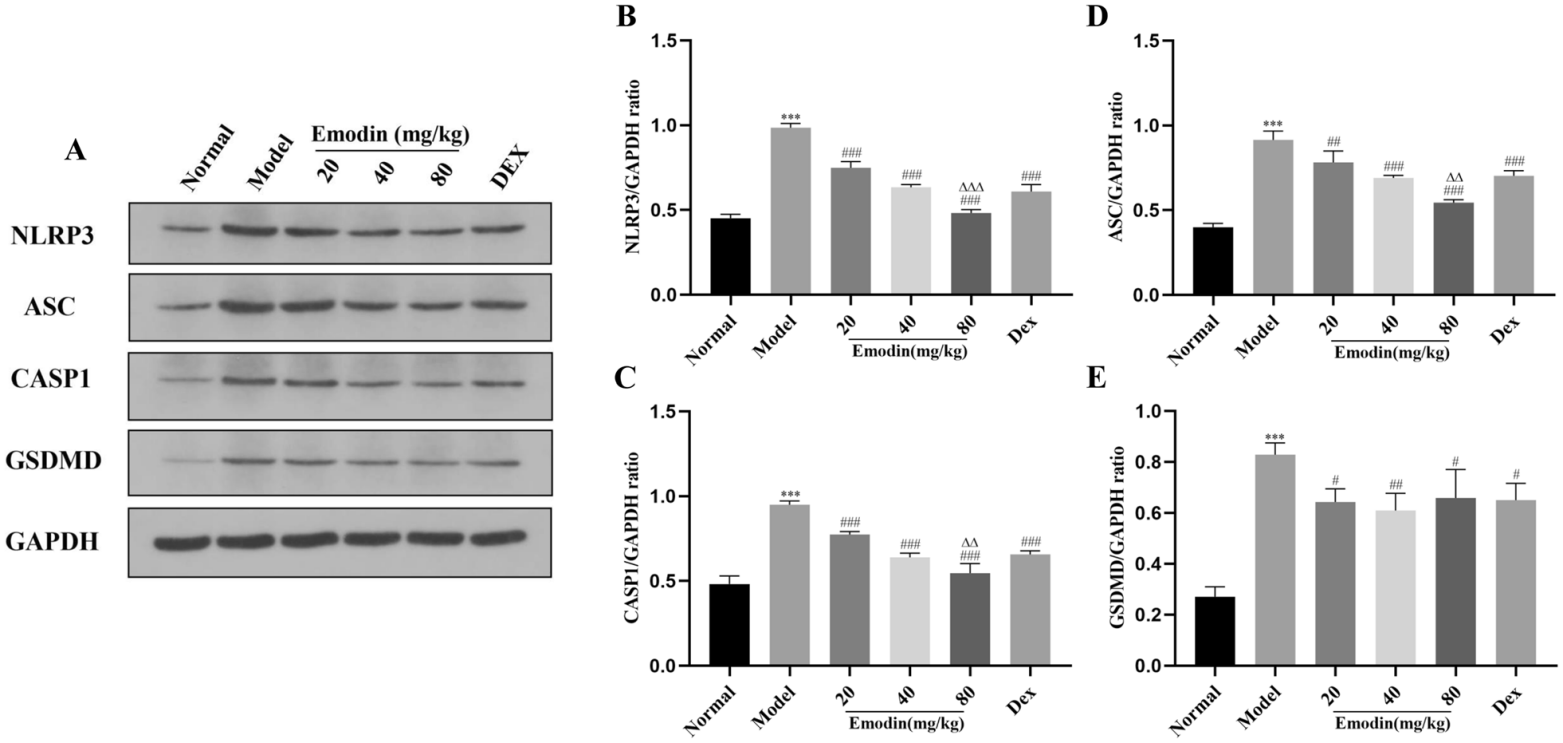
Effects of emodin on NLRP3 inflammasome-dependent signaling pathway-associated proteins in a rat model. **A**–**E** The protein levels of NLRP3, ASC, CASP1, and GSDMD were detected by western blotting, and the grayscale values of the bands were analyzed using ImageJ software. All data are presented as the mean ± SD (*n* = 8). ^***^P < 0.001 vs. normal group. ^#^*P* < 0.05, ^##^*P* < 0.01, ^###^*P* < 0.001 vs. model group; ^∆∆^*P* < 0.01, ^∆∆∆^*P* < 0.001 vs. DEX group.

**Fig. 8:**
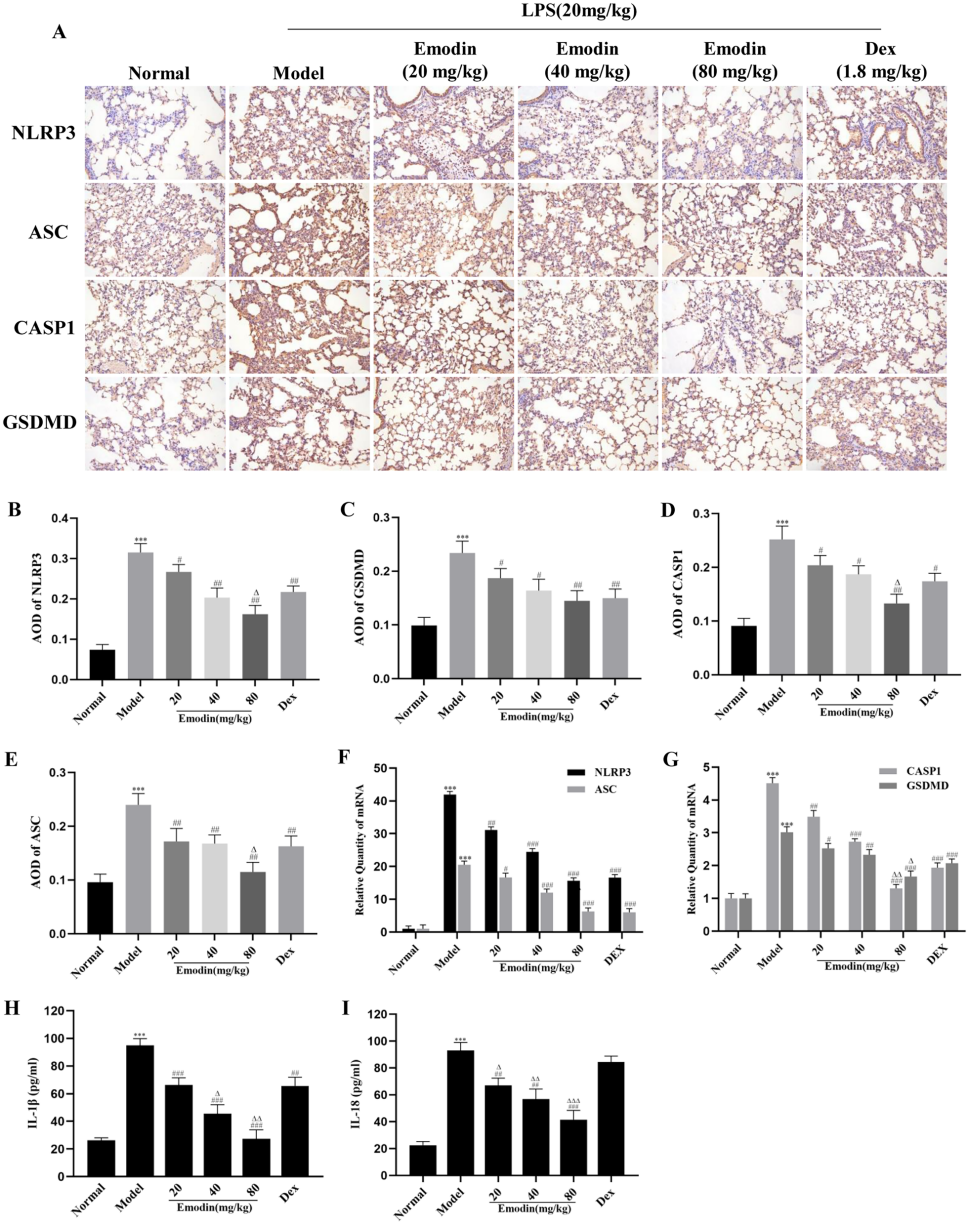
Effects of emodin on NLRP3 inflammasome-dependent signaling pathway in rat lung tissues. **A** Semi-quantitative evaluation of NLRP3, CASP1, ASC, and GSDMD immunohistochemical staining (original magnification 200 ×). **B**–**E** The average OD was determined by Image-Pro Plus 6.0 software. **F**–**G** The mRNA levels of NLRP3, CASP1, ASC, and GSDMD in rat lung tissues were determined by RT-PCR. **H**–**I** Effects of emodin on the expression of IL-1β and IL-18 in rat serum was determined by ELISA. Data are shown as the mean ± SD (*n* = 8). ^***^P < 0.001 vs. normal group; ^#^*P* < 0.05, ^##^*P* < 0.01, ^###^*P* < 0.001 vs. model group; ^Δ^*P* < 0.05, ^ΔΔ^*P* < 0.01, ^ΔΔΔ^*P* < 0.001 vs. DEX group.

As shown in Figs. 7A-E and 8A-G, compared with the normal group, the mRNA and protein levels of NLRP3, CASP1, ASC, and GSDMD in the model group were markedly increased. The levels of NLRP3 and downstream molecules in the emodin and DEX groups were decreased compared with those in the model group. Moreover, the ASC and GSDMD mRNA levels in the 80 mg/kg emodin group; the CASP1 mRNA levels in the 80 mg/kg emodin group; and the NLRP3, CASP1, and ASC protein levels in the 80 mg/kg emodin group were markedly lower than those in the DEX group. These results showed that emodin significantly reduced NLRP3 and expression of its downstream molecules in rat lung tissues after LPS stimulation, similar to or better than the effects of DEX.

### Effects of Emodin on IL-1β and IL-18 Levels in LPS-Induced ALI

The expression levels of IL-1β and IL-18 in rat serum were detected by ELISA. As shown in Fig. 8H, I, the levels of IL-1β and IL-18 in the model group were notably increased compared with those in the normal group. Compared with the model group, IL-1β and IL-18 levels were markedly reduced in the 20, 40, and 80 μg/mL emodin groups and DEX group. In addition, emodin (20, 40, 80 μg/mL) had stronger inhibitory effects on IL-18 than DEX; however, only emodin (40, 80 μg/mL) showed stronger inhibitory effects than DEX. These results showed that emodin had a marked effect on inhibiting the production of IL-1β and IL-18 in LPS-induced ALI.

## DISCUSSION

ARDS is a common life-threatening disease with a poor prognosis, which can be caused by a variety of factors such as systemic inflammation, sepsis, and pneumonia [2]. LPS has been widely used to establish inflammatory response models [16]. During the progression of ALI, excessive inflammatory reactions aggravate the damage to bodies, so it is extremely important to discover early potent interventions to control lung injury development. DEX has anti-inflammatory [17], immunosuppressive [18], and other pharmacological effects. Although DEX can effectively inhibit inflammation and alleviate the damage of organs, it cannot be used for a long period of time, as the long-term use of glucocorticoids leads to many side effects such as weight gain, osteoporosis, and cataract [19]. Emodin, a natural product isolated from rhubarb, exerts anti-inflammatory and antioxidant effects. In this study, we observed the protective effects and explored the mechanisms of emodin in LPS-induced ALI *in vitro* and *in vivo*.

Pathological changes of ALI are usually characterized by pulmonary edema, lung, and alveolar hemorrhage, and inflammatory cell infiltration. Our results showed that emodin significantly ameliorated the pathologic changes in the lung tissues compared with the LPS group. Oxidative stress caused by excessive ROS can lead to severe inflammation of the lungs, which in turn triggers ALI/ARDS [20]. MPO is a marker of neutrophil accumulation, which is generated from neutrophils [21]. In the state of inflammation, it is released into the extracellular fluid and is involved in the oxidation of lipids, which may further lead to tissue damage [22]. MDA, a product of lipid peroxidation, is a marker of oxidative stress. In our study, we found that emodin can suppress the production of ROS, MPO, and MDA. These results suggest that emodin has protective effects on LPS-induced ALI through antioxidation and emodin may inhibit the expression of NLRP3 by decreasing the production of ROS.

The NLRP3 inflammasome is a protein complex composed of NLRP3, pro-caspase-1, and ASC, which plays a role in sensing microbes and many endogenous danger signals such as bacterial toxin and fibrillary amyloid-β peptide [23]. Assembly of the NLRP3 inflammasome leads pro-CASP1 to mature to CASP1. Then mature caspase-1 activates GSDMD to form holes in the cell membrane, which aggravates the release of IL-1β and IL-18 and then triggers a more serious inflammatory response [4]. Activation of the NLRP3 inflammasome accelerates the progress of inflammation in ALI [24]. GSDMD, the pore-forming protein in pyroptosis, can be activated by pro-inflammatory caspases and form membrane pores, which cause membrane rupture and the release of cellular contents such as IL-1β and IL-18 [25]. IL-1β and IL-18, as products of cell pyroptosis, play important roles in the development of ALI, which can trigger inflammatory cascades and aggravate the inflammatory response [26]. In addition, the excessive release of IL-1β can increase the permeability of alveolar epithelial and vascular endothelial cells, which will lead to pulmonary edema [27]. The expression of IL-18 is negatively correlated with the long-term survival of patients with ARDS [28]. Thus, the NLRP3 inflammasome-dependent pyroptosis signaling pathway may be a potentially therapeutic target in LPS-induced ALI.

In this study, we demonstrated that emodin was able to suppress the NLRP3 inflammasome-dependent pyroptosis *in vitro* and *in vivo*. *In vitro*, the expression levels of NLRP3, CASP1, ASC, GSDMD, IL-1β, and IL-18 were significantly inhibited by emodin. We used the siRNA technique to knockdown NLRP3 in J774A.1 cells. We observed that emodin decreased the expression of NLRP3, and then inhibited the expression of CASP1, ASC, GSDMD, IL-1β, and IL-18. *In vivo*, we confirmed the inhibitory effects of emodin on NLRP3 and its downstream molecules. The abovementioned results showed that emodin has protective effects on LPS-induced ALI by inhibiting NLRP3 inflammasome-dependent pyroptosis.

In conclusion, this study demonstrated that emodin can alleviate LPS-induced ALI by inhibiting the NLRP3 inflammasome-dependent pyroptosis signaling pathway *in vitro* and *in vivo*. Therefore, emodin may be a potential and therapeutic approach for the treatment of ALI. Future studies should evaluate the clinical relevance of these experimental findings.

## Data Availability

All data generated or analyzed during this study are included in this published article.

## References

[1] Butt Y, Kurdowska A, Allen TC (2016). Acute lung injury: A clinical and molecular review. Archives of pathology & laboratory medicine.

[2] Ware LB, Matthay MA (2000). The acute respiratory distress syndrome. The New England journal of medicine.

[3] Ding J, Wang K, Liu W, She Y, Sun Q, Shi J (2016). Pore-forming activity and structural autoinhibition of the gasdermin family. Nature.

[4] He Y, Hara H, Nunez G (2016). Mechanism and regulation of NLRP3 inflammasome activation. Trends in biochemical sciences.

[5] Wang S, Zhao J, Wang H, Liang Y, Yang N, Huang Y (2015). Blockage of P2X7 attenuates acute lung injury in mice by inhibiting NLRP3 inflammasome. International immunopharmacology.

[6] Shi X, Xie WL, Kong WW, Chen D, Qu P (2015). Expression of the NLRP3 Inflammasome in Carotid Atherosclerosis. Journal of stroke and cerebrovascular diseases : The official journal of National Stroke Association.

[7] Chen X, Liu G, Yuan Y, Wu G, Wang S, Yuan L (2019). NEK7 interacts with NLRP3 to modulate the pyroptosis in inflammatory bowel disease via NF-kappaB signaling. Cell death & disease.

[8] Ying Y, Mao Y, Yao M (2019). NLRP3 Inflammasome Activation by MicroRNA-495 promoter methylation may contribute to the progression of acute lung injury. Molecular therapy Nucleic acids.

[9] Zhou J, Li G, Han G, Feng S, Liu Y, Chen J (2020). Emodin induced necroptosis in the glioma cell line U251 via the TNF-alpha/RIP1/RIP3 pathway. Investigational new drugs.

[10] Dong Y, Zhang L, Jiang Y, Dai J, Tang L, Liu G (2019). Emodin reactivated autophagy and alleviated inflammatory lung injury in mice with lethal endotoxemia. Experimental animals.

[11] Xiao M, Zhu T, Zhang W, Wang T, Shen YC, Wan QF (2014). Emodin ameliorates LPS-induced acute lung injury, involving the inactivation of NF-κB in mice. International journal of molecular sciences.

[12] Yang F, Wang Y, Li G, Xue J, Chen ZL, Jin F (2018). Effects of corilagin on alleviating cholestasis via farnesoid X receptor-associated pathways *in vitro* and *in vivo*. British journal of pharmacology.

[13] Ding Y, Liu P, Chen ZL, Zhang SJ, Wang YQ, Cai X (2018). Emodin Attenuates Lipopolysaccharide-Induced Acute Liver Injury via Inhibiting the TLR4 Signaling Pathway *in vitro* and *in vivo*. Frontiers in pharmacology.

[14] Martinon F (2010). Signaling by ROS drives inflammasome activation. European journal of immunology.

[15] Mittal M, Siddiqui MR, Tran K, Reddy SP, Malik AB (2014). Reactive oxygen species in inflammation and tissue injury. Antioxidants & redox signaling.

[16] Matute-Bello G, Frevert CW, Martin TR (2008). Animal models of acute lung injury. American journal of physiology Lung cellular and molecular physiology.

[17] Bartneck, M., F.M. Peters, K.T. Warzecha, M. Bienert, L. van Bloois, C. Trautwein, et al. 2014. Liposomal encapsulation of dexamethasone modulates cytotoxicity inflammatory cytokine response and migratory properties of primary human macrophages. *Nanomedicine: nanotechnology biology and medicine* 10: 1209–1220. 10.1016/j.nano.2014.02.011 24607939

[18] Giles AJ, Hutchinson MND, Sonnemann HM, Jung J, Fecci PE, Ratnam NM (2018). Dexamethasone-induced immunosuppression: Mechanisms and implications for immunotherapy. Journal for immunotherapy of cancer.

[19] Oray M, Abu Samra K, Ebrahimiadib N, Meese H, Foster CS (2016). Long-term side effects of glucocorticoids. Expert opinion on drug safety.

[20] Quinlan GJ, Lamb NJ, Tilley R, Evans TW, Gutteridge JM (1997). Plasma hypoxanthine levels in ARDS: Implications for oxidative stress morbidity and mortality. American journal of respiratory and critical care medicine.

[21] Klebanoff SJ (2005). Myeloperoxidase: Friend and foe. Journal of leukocyte biology.

[22] Kisic B, Miric D, Dragojevic I, Rasic J, Popovic L (2016). Role of myeloperoxidase in patients with chronic kidney disease. Oxidative medicine and cellular longevity.

[23] Martinon F, Mayor A, Tschopp J (2009). The inflammasomes: Guardians of the body. Annual review of immunology.

[24] Hosseinian N, Cho Y, Lockey RF, Kolliputi N (2015). The role of the NLRP3 inflammasome in pulmonary diseases. Therapeutic advances in respiratory disease.

[25] Shi J, Zhao Y, Wang K, Shi X, Wang Y, Huang H (2015). Cleavage of GSDMD by inflammatory caspases determines pyroptotic cell death. Nature.

[26] Goodman RB, Pugin J, Lee JS, Matthay MA (2003). Cytokine-mediated inflammation in acute lung injury. Cytokine & growth factor reviews.

[27] Hybertson BM, Lee YM, Cho HG, Cho OJ, Repine JE (2000). Alveolar type II cell abnormalities and peroxide formation in lungs of rats given IL-1 intratracheally. Inflammation.

[28] Makabe H, Kojika M, Takahashi G, Matsumoto N, Shibata S, Suzuki Y (2012). Interleukin-18 levels reflect the long-term prognosis of acute lung injury and acute respiratory distress syndrome. Journal of anesthesia.

